# Digital therapeutics and mHealth applications in cardiovascular prevention: strongest evidence in hypertension and emerging perspectives in dyslipidemia

**DOI:** 10.3389/fdgth.2026.1915478

**Published:** 2026-09-02

**Authors:** Arkadiusz Wysocki, Małgorzata Wierzowiecka, Arkadiusz Niklas

**Affiliations:** 1 Department of Hypertension, Angiology and Internal Medicine, Poznan University of Medical Sciences, Poznan, Poland; 2 Institute of Neurological Disorders, Poznan University of Medical Sciences, Poznań, Poland

**Keywords:** cardiovascular prevention, digital health ecosystems, digital therapeutics, dyslipidemia, hypertension, mHealth, telemonitoring

## Abstract

Digital health interventions are increasingly used to support cardiovascular prevention, but their clinical maturity differs across risk factors and types of technology. This narrative review summarizes current evidence and implementation challenges related to digital therapeutics, mobile health applications, telemonitoring systems, and integrated digital health platforms in hypertension, dyslipidemia, and broader cardiometabolic prevention. The strongest evidence currently supports digitally enabled blood pressure management, particularly when home blood pressure monitoring, telemonitoring, behavioral support, medication adherence tools, and clinician-guided treatment adjustment are integrated into routine care. In contrast, digital interventions for dyslipidemia remain less established and are mainly focused on education, lifestyle modification, adherence support, shared decision-making, and long-term risk reduction. Therefore, most lipid-related digital tools should currently be interpreted as supportive mHealth or prevention interventions rather than fully established digital therapeutics. Successful implementation requires more than patient-facing applications. It depends on clinical workflow integration, professional responsibility for data review and telemonitoring alerts, regulatory and reimbursement pathways, data protection, interoperability, equity, and evidence of clinical and economic value. Future studies should distinguish between digital therapeutics, mHealth tools, telemonitoring systems, and digital ecosystems, and should evaluate clinically meaningful endpoints, safety, cost-effectiveness, and long-term sustainability.

## Introduction

Cardiovascular diseases (CVD) remain the leading cause of mortality in Poland and across Europe, accounting for approximately 40%–45% of all deaths (1, 2).

Among them, myocardial infarction, stroke, and heart failure are particularly important, sharing common traditional risk factors such as hypertension, dyslipidemia, tobacco smoking, obesity, unhealthy diet, and physical inactivity (3, 4). Despite substantial advances in pharmacotherapy and the widespread availability of evidence-based clinical guidelines, the control of these risk factors in the Polish population remains suboptimal.

Hypertension continues to be the most prevalent single cardiovascular risk factor. In the WOBASZ II study, which included adult Polish residents aged 19–99 years, the overall prevalence of hypertension was 42.7%. The prevalence was significantly higher in men than in women (46.2% vs. 40.4%). Effective blood pressure control was achieved in only 23% of the entire cohort. Women demonstrated higher rates of blood pressure normalization compared with men (27.3% vs. 19.0%) (5).

Dyslipidemia represents the second most common cardiovascular risk factor. In the WOBASZ II study, hypercholesterolemia (total cholesterol ≥190 mg/dL or LDL cholesterol ≥115 mg/dL) was present in 70.3% of men and 64.3% of women. Notably, 60.6% of affected individuals were unaware of their condition, with no significant sex-related differences. An additional 17% were aware of their diagnosis but remained untreated, either pharmacologically or through dietary interventions. Effective cholesterol control was achieved in less than 6% of the population (6, 7).

Overweight and obesity constitute another major public health challenge. In WOBASZ II, obesity (body mass index [BMI] ≥ 30 kg/m^2^) was identified in 24.4% of men and 25.0% of women, whereas overweight (BMI 25.0–29.9 kg/m^2^) was present in 43.2% and 30.5%, respectively. During the decade (2005–2015), the prevalence of abdominal obesity increased among men from 26.8% (95% CI, 25.6–28.1) to 30.7% (95% CI, 28.6–32.8), and among women from 40.2% (95% CI, 38.7–41.6) to 42.2% (95% CI, 40.0–44.5) (8).

Tobacco smoking remains an important cardiovascular risk factor, although its prevalence has declined in recent years. In WOBASZ II, regular smoking was reported by 29.9% of men and 20.5% of women, while an additional 3%–4% reported occasional smoking (9). This corresponds to nearly eight million adult smokers in Poland.

Physical activity levels in the Polish population remain low. During a decade, the proportion of individuals meeting the minimum World Health Organization (WHO) recommendations for physical activity decreased from 37.4% to 27.3% among men and from 32.7% to 28.3% among women. Physical inactivity is particularly common among older adults and residents of large urban areas (10).

**Figure 1:**
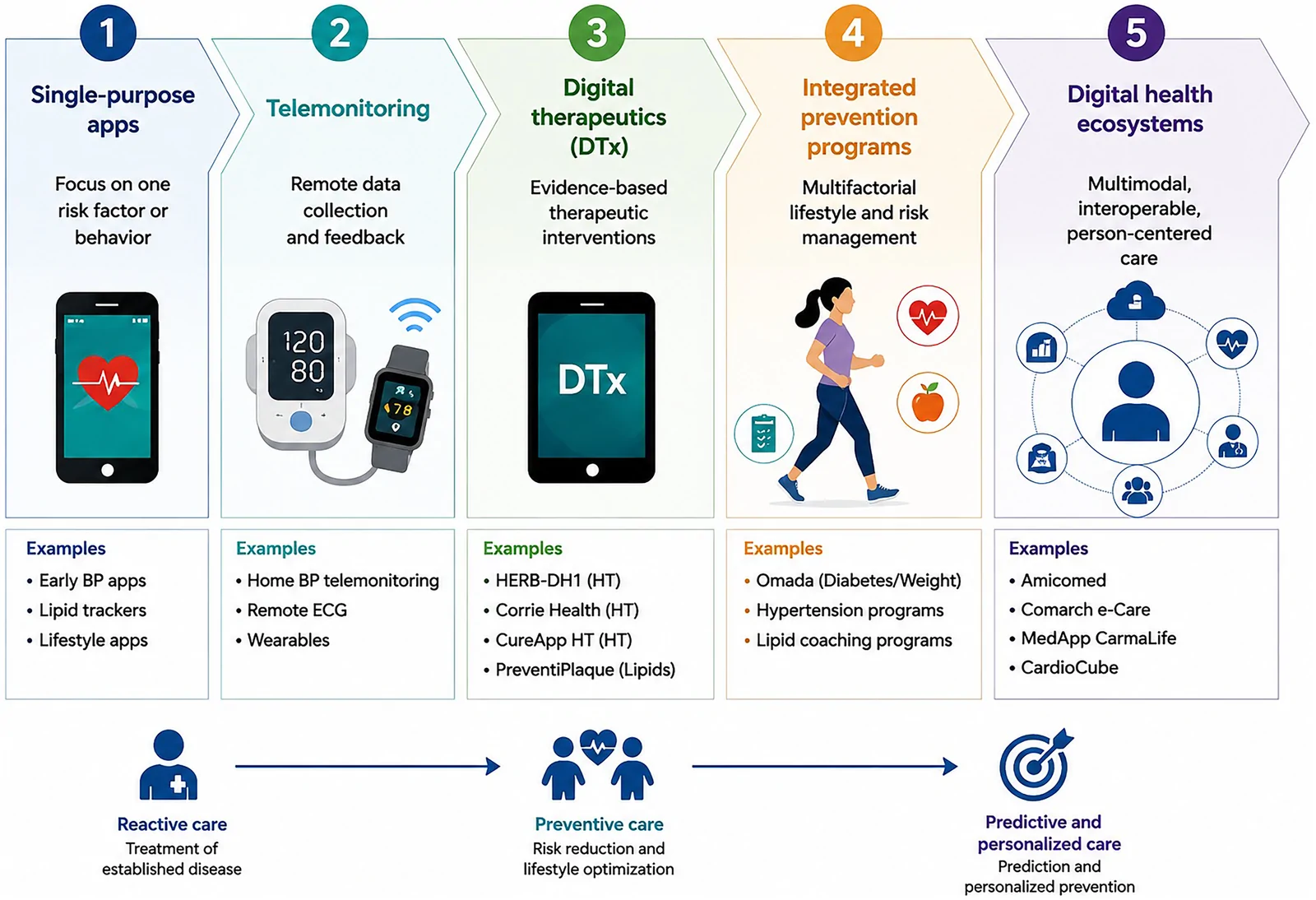
Evolution of cardiovascular digital health: from single-purpose applications to integrated digital ecosystems. BP, blood pressure; DTx, digital therapeutics; HT, hypertension; ECG, electrocardiography.

Taken together, the Polish population is characterized by a high prevalence of traditional cardiovascular risk factors and unsatisfactory levels of risk-factor control. These observations underscore the need for novel tools capable of supporting both healthcare systems and individual patients in achieving long-term risk-factor management. In this context, rapidly evolving digital health solutions—including mobile applications, telemedicine platforms, and digital therapeutics (DTx)—are gaining increasing importance. The evolution of cardiovascular digital health from single-purpose applications to integrated digital ecosystems is summarized in Figure 1.

### Search strategy and source selection

This article was designed as a narrative review, not as a systematic review or meta-analysis. Its aim was to provide a clinically oriented and implementation-focused synthesis of digital therapeutics, mHealth applications, telemonitoring systems, and integrated digital health platforms relevant to hypertension, dyslipidemia, and cardiovascular prevention.

A structured literature search was performed in PubMed and Scopus. The search was supplemented by manual screening of reference lists from relevant reviews, clinical trials, guidelines, consensus documents, and implementation reports. In addition, selected regulatory, reimbursement, and institutional sources were reviewed, including documents and publicly available information from regulatory agencies, reimbursement frameworks, and official digital health programme websites. The search included publications and documents available up to June 2026.

The search strategy combined terms related to digital health technologies and cardiovascular prevention. Key concepts included: “digital therapeutics”, “digital health”, “mHealth”, “mobile health applications”, “telemonitoring”, “remote monitoring”, “home blood pressure monitoring”, “hypertension”, “blood pressure control”, “dyslipidemia”, “lipid management”, “cardiovascular prevention”, “medication adherence”, “behavioral intervention”, “clinical decision support”, “digital health platform”, “digital ecosystem”, “implementation”, “certification”, “reimbursement”, “DiGA”, “Medical Device Regulation”, “MDR”, “FDA”, and “cost-effectiveness”.

Eligible sources included randomized clinical trials, observational studies, real-world studies, implementation studies, systematic reviews, meta-analyses, clinical guidelines, consensus statements, regulatory documents, reimbursement frameworks, and selected grey literature from official institutional or programme websites. Sources were prioritized if they provided information on clinical effectiveness, blood pressure or lipid-related outcomes, adherence, patient engagement, implementation feasibility, regulatory status, reimbursement pathways, cost-effectiveness, workflow integration, or safety and data governance.

Examples of digital interventions and platforms were selected for discussion when they were clinically relevant to hypertension, dyslipidemia, or broader cardiometabolic prevention; when they had published evidence or documented implementation experience; or when they illustrated important regulatory, reimbursement, or ecosystem-level aspects of digital cardiovascular care. Digital tools focused exclusively on general wellness without a defined medical or preventive objective were not included. Sources with insufficient methodological detail, unclear clinical relevance, or purely promotional content were excluded.

**Figure 2:**
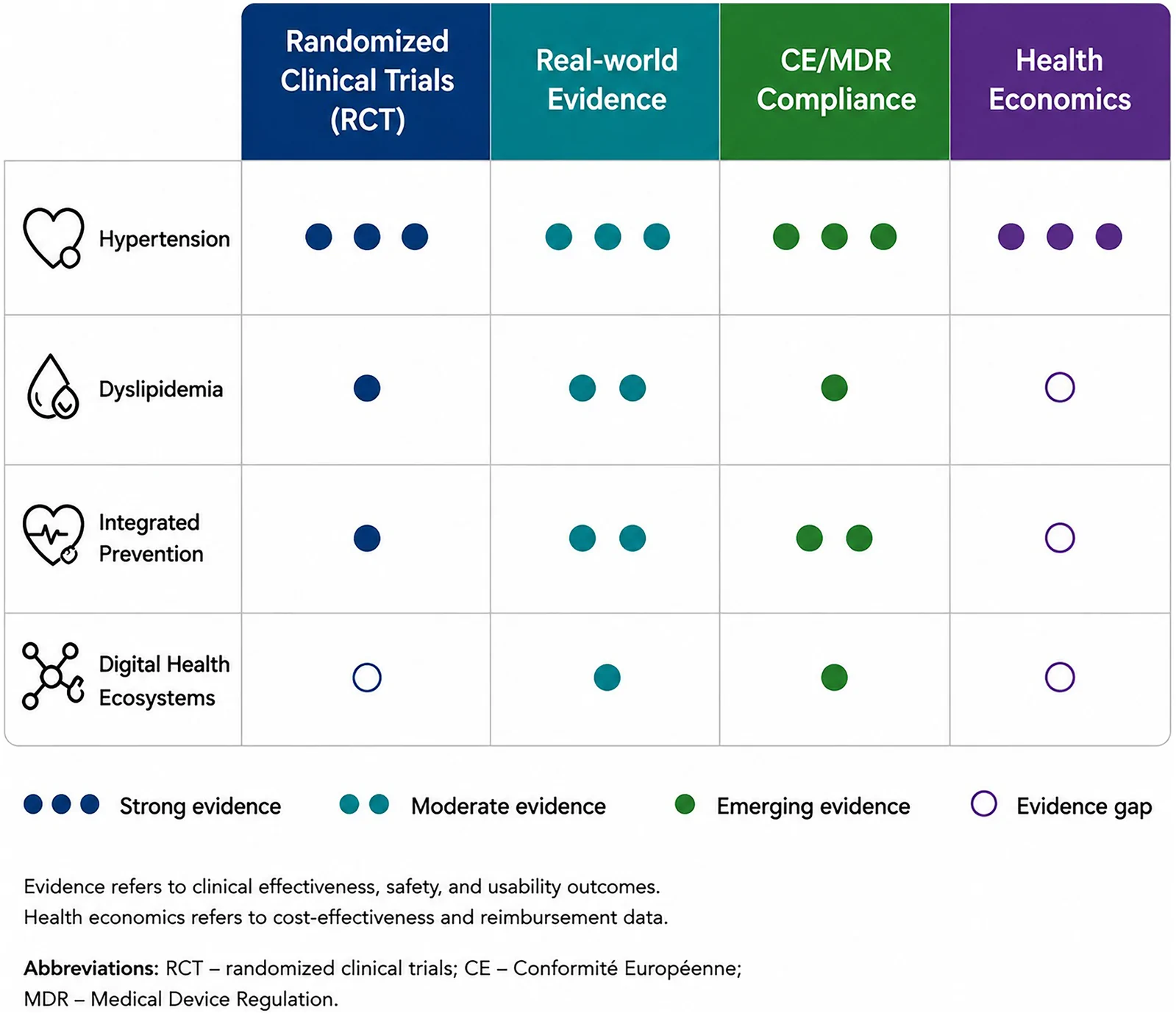
Current evidence landscape in cardiovascular digital therapeutics. ●●● strong evidence; ●● moderate evidence; ● emerging evidence; ○ evidence gap. RCT, randomized clinical trial; CE, *Conformité Européenne*; MDR, Medical Device Regulation.

Because of the narrative scope of this review, no formal risk-of-bias assessment, protocol registration, or quantitative evidence synthesis was performed. Instead, the evidence was synthesized narratively, with attention to the type of intervention, clinical indication, level of evidence, implementation maturity, regulatory and reimbursement status, and relevance to routine cardiovascular prevention. Particular emphasis was placed on distinguishing established evidence from emerging or preliminary evidence, especially when comparing digital interventions for hypertension with those for dyslipidemia and integrated cardiovascular prevention**.** The current evidence landscape across the main categories of cardiovascular digital interventions is summarized in Figure 2.

### Terminology and classification of digital cardiovascular interventions

The terminology used in digital cardiovascular health is heterogeneous, and several terms are often used interchangeably, including digital therapeutics, mHealth applications, telemonitoring systems, digital health platforms, and integrated digital ecosystems. However, these categories differ substantially in their therapeutic intent, level of clinical evidence, regulatory requirements, reimbursement pathways, and degree of integration into routine care.

In this review, digital therapeutics are defined as evidence-based software-driven interventions designed to prevent, manage, or treat a medical condition. Digital therapeutics usually deliver structured therapeutic content, behavioral interventions, algorithm-guided support, or clinical decision pathways. Depending on the jurisdiction and intended use, they may require certification as a medical device, regulatory authorization, prescription, or inclusion in a reimbursement framework.

mHealth applications are defined more broadly as mobile or web-based tools that support health-related behaviors, education, self-monitoring, lifestyle modification, medication adherence, or patient engagement. Although such applications may be clinically useful, they should not automatically be considered digital therapeutics unless they make a specific therapeutic claim and are supported by appropriate clinical evidence and regulatory status.

Telemonitoring systems refer to digital solutions that enable remote collection, transmission, and review of physiological or clinical data, such as blood pressure, heart rate, body weight, symptoms, physical activity, or laboratory-related information. Telemonitoring may facilitate clinical decision-making and treatment adjustment, but it does not necessarily constitute a digital therapeutic unless it includes a validated therapeutic intervention, structured behavioral programme, or algorithm-driven care pathway.

**Figure 3:**
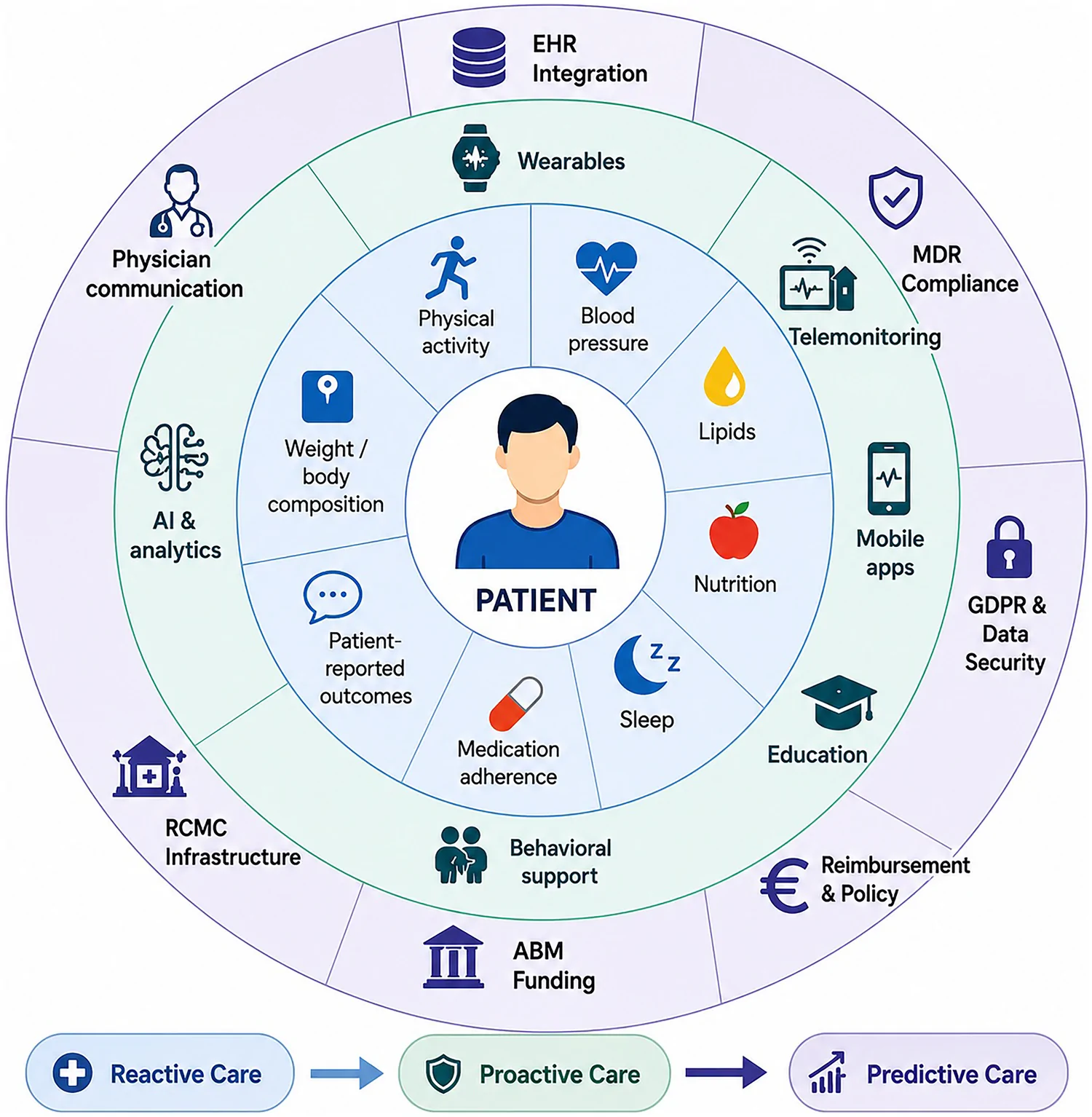
Conceptual framework of integrated cardiovascular digital health ecosystems. EHR, electronic health record; MDR, Medical Device Regulation; GDPR, General Data Protection Regulation; ABM, Agency for Medical Research (Poland); RCMC, Regional Centres for Digital Medicine (Poland).

Digital health platforms are broader infrastructures that integrate multiple components, including patient-facing applications, connected devices, clinician dashboards, alerts, education modules, adherence tools, and sometimes clinical decision support. Integrated digital health ecosystems represent a still broader model in which patient-generated health data, healthcare professionals, electronic health records, reimbursement mechanisms, regulatory governance, and longitudinal care pathways are connected within a coordinated system of care (31). A conceptual framework of an integrated cardiovascular digital health ecosystem is presented in Figure 3.

This distinction is clinically important because the level of evidence and implementation maturity differs substantially between these categories. In the present review, selected examples are therefore classified according to their primary function and implementation maturity rather than being uniformly labelled as digital therapeutics. The main categories and characteristics of the digital cardiovascular interventions discussed in this review are summarized in Table 1.

**Table 1:** Classification of selected digital cardiovascular interventions discussed in the review.

Intervention type/example	Main category	Clinical indication	Primary function	Evidence level	Regulatory status	Reimbursement status	Implementation context
Structured software-based intervention for blood pressure control	Digital therapeutic	Hypertension	Behavioral intervention, self-management, treatment support	Randomized or real-world clinical evidence, where available	May require certification as medical device/DTx depending on jurisdiction	Variable; reimbursed in selected systems	Clinical or hybrid care pathway
Home blood pressure telemonitoring program	Telemonitoring system	Hypertension	Remote BP transmission, professional review, treatment adjustment	Relatively strong evidence, especially when combined with clinician-guided care	Device- and software-dependent	Variable; often program-based	Primary care, specialist care, telemedicine
General blood pressure tracking application	mHealth application	Hypertension	Self-monitoring, reminders, education, patient engagement	Variable; often observational or usability-based	Usually not certified as DTx	Usually not reimbursed	Patient self-management
Medication adherence or lifestyle application for lipid management	mHealth application	Dyslipidemia	Education, reminders, lifestyle support, adherence support	Preliminary; often feasibility, adherence, or behavioral outcomes	Usually not certified as DTx	Usually not reimbursed	Prevention and chronic disease support
Integrated cardiovascular prevention platform	Digital health platform	Hypertension, dyslipidemia, cardiometabolic risk	Multimodal monitoring, education, adherence support, clinician dashboard	Emerging; implementation or real-world evidence	Variable	Variable; often local or pilot -based	Integrated prevention program
AI-assisted clinical decision support tool	Digital health software/decision support	Cardiovascular prevention	Risk stratification, treatment suggestions, prioritization of care	Emerging; requires prospective validation	Depends on intended use and jurisdiction	Variable	Clinician-facing decision support
Nationally listed or reimbursed digital health application	Certified digital health application/DTx, depending on function	Condition-specific	Structured digital intervention or disease management support	Evidence required by national framework	Certified or listed under national pathway	Reimbursed under defined criteria	National digital health program

### Digital therapeutics for hypertension: evidence from randomized clinical trials

Hypertension remains the most common modifiable cardiovascular risk factor. Despite the availability of effective antihypertensive medications, fixed-dose combination therapies, and regularly updated guidelines issued by the European Society of Cardiology (ESC), the European Society of Hypertension (ESH), and the Polish Society of Hypertension (PTNT), fewer than one in four patients with diagnosed hypertension achieve recommended blood pressure targets (12). Poor treatment adherence and insufficient long-term monitoring in routine clinical practice remain among the major barriers to effective blood pressure control.

A landmark study in the field of digital therapeutics was the HERB-DH1 trial conducted in Japan, which enrolled nearly 400 patients with essential hypertension randomized 1:1 to either a digital therapeutics intervention (HERB system plus standard lifestyle modification) or standard lifestyle modification alone. The HERB system consisted of a mobile application, a cloud-based platform, and a physician dashboard, providing patients with personalized lifestyle recommendations and reminders regarding blood pressure measurements. The study demonstrated a significant reduction in 24 h ambulatory systolic blood pressure compared with the control group, accompanied by improved adherence to lifestyle recommendations (13, 14).

Additional evidence supporting app-based blood pressure management originates from the SMART-BP study, which evaluated the effectiveness of self-monitoring combined with structured feedback delivered through a mobile application among patients with uncontrolled hypertension. The intervention was associated with improved blood pressure control and high patient engagement, highlighting the potential value of simple digital tools integrated into routine care. Although the study was smaller and less comprehensive than HERB-DH1, its findings further support the role of mHealth interventions in hypertension management (15).

Additional evidence comes from a randomized clinical trial evaluating a WeChat-based multimodal digital transformation management model in patients with new-onset mild-to-moderate hypertension. The intervention significantly improved home and office blood pressure control compared with usual care and was associated with favourable changes in selected quality-of-life measures. This study supports the concept that multimodal mHealth-based care models may enhance blood pressure management when patient engagement, monitoring, and individualized feedback are combined within a single digital intervention (16).

### European and Polish solutions certified as medical devices

The Amicomed application (Newel Health, Italy) was among the first digital health solutions in Europe to obtain certification under the Medical Device Regulation (MDR) as a Class IIa medical device. The platform enables blood pressure data analysis, provides personalized recommendations, and integrates with telemedicine systems (17, 18).

Several innovative solutions are also being developed and implemented in Poland:

- **Comarch e-Care**—a certified telemedicine platform designed for remote monitoring of patients with chronic diseases. The system combines a mobile application with connected medical monitoring devices, enabling continuous home-based care. The set of monitoring devices can be tailored to individual patient needs (19).
- **MedAppAI CarnaLife System**—a digital platform integrating medical devices and artificial intelligence (AI)-based algorithms, currently used in several healthcare networks, including Medicover (20).
- **CardioCube**—a voice-assisted telemedicine system supporting patients with heart failure and hypertension. Studies have demonstrated high acceptance and willingness among patients with cardiovascular disease (CVD) to use modern voice-based telemedicine solutions, including voice conversational agents combined with physician support delivered via telephone consultations (21).

### Applications supporting healthcare professionals

In addition to patient-oriented solutions, a growing number of digital tools are designed to support healthcare professionals in individualizing treatment. These platforms promote evidence-based pharmacotherapy and provide reliable information facilitating clinical decision-making.

Examples include:

- **mniejlekow.pl**—an educational platform and mobile application developed under the initiative of the Polish Society of Hypertension (PTNT), aimed at supporting both physicians and patients in treatment optimization and rational pharmacotherapy.
- **Medycyna Praktyczna**—a widely used professional medical platform providing continuously updated clinical guidelines, expert lectures, and evidence-based recommendations for contemporary clinical practice.
- **MediPlanner**—a platform currently under development that supports physicians in the diagnosis and management of hypertension and lipid disorders. The system integrates with Hospital Information Systems (HIS) and Electronic Medical Records (EMR), extracting clinical data for analysis and assisting physicians in developing individualized treatment plans.
- **Hypertension Guidelines (Nadciśnienie Tętnicze)**—a mobile application providing rapid access to current recommendations and treatment algorithms issued by major scientific societies, including the European Society of Cardiology (ESC).

### Cost-effectiveness

Economic evaluation is increasingly important for the implementation of digital therapeutics and telemonitoring in cardiovascular care. In hypertension, available analyses suggest that digitally supported care may be cost-effective in selected models, particularly when reductions in blood pressure are translated into projected decreases in long-term cardiovascular events. For example, economic modelling based on the HERB-DH1 trial suggested potential cost-effectiveness of a digital therapeutic intervention for essential hypertension, while European analyses of self-monitoring and telemonitoring strategies have also reported favourable model-based economic outcomes (22, 23).

However, these findings should be interpreted cautiously. Modeled economic benefits do not necessarily indicate empirically demonstrated cost savings in routine clinical practice. Real-world cost-effectiveness depends on healthcare system structure, reimbursement mechanisms, patient adherence, clinician workload, technical support, integration with electronic health records, and long-term sustainability. Therefore, future studies should evaluate not only clinical outcomes but also actual healthcare utilization, staff time, implementation costs, and sustained economic value in real-world care pathways.

### Current limitations and challenges

Despite the growing body of clinical evidence supporting digital therapeutics, several important challenges remain:

- Heterogeneity of study designs and a lack of standardized clinical endpoints;
- relatively short follow-up periods, typically ranging from 3 to 6 months;
- Difficulties in maintaining long-term patient engagement and adherence to digital interventions;
- Barriers related to the use of digital technologies among older adults;
- Technical challenges, including device costs, internet connectivity, and technical support;
- limited integration with Electronic Medical Record (EMR) systems;
- The absence of clear reimbursement pathways for digital therapeutics in most European countries, with Germany's Digital Health Applications (DiGA) framework representing one of the few notable exceptions.

### Digital support for lipid management: current evidence

Digital tools in dyslipidemia: an emerging but less mature evidence base

Compared with hypertension, the evidence base for digital interventions in dyslipidemia remains less mature. While digitally enabled blood pressure management has been evaluated in multiple randomized trials, meta-analyses, telemonitoring programs, and real-world care models, lipid-focused digital interventions are more often limited to feasibility studies, adherence-support tools, lifestyle applications, patient education, shared decision-making aids, or broader cardiovascular prevention programs.

Most currently available digital tools in dyslipidemia aim to improve medication adherence, promote lifestyle modification, support patient engagement, or facilitate long-term risk communication rather than directly deliver a validated therapeutic intervention with demonstrated lipid-lowering efficacy. Therefore, such tools should generally be classified as supportive mHealth or cardiovascular prevention interventions rather than established digital therapeutics, unless they have shown clinically meaningful effects on lipid parameters, adherence, treatment intensification, or cardiovascular outcomes in appropriately designed studies.

This distinction is important for both clinical interpretation and implementation. In hypertension, remote monitoring and clinician-guided treatment adjustment can be directly linked to measurable blood pressure reduction. In dyslipidemia, however, the relationship between digital engagement, adherence behavior, lipid lowering, and long-term cardiovascular outcomes is usually more indirect and requires longer follow-up. Consequently, claims regarding digital therapeutics in dyslipidemia should remain cautious, and future studies should prioritize randomized or pragmatic real-world designs assessing LDL-C reduction, treatment persistence, therapeutic intensification, achievement of guideline-recommended lipid targets, and long-term cardiovascular risk reduction.

Dyslipidemia, particularly elevated low-density lipoprotein cholesterol (LDL-C), remains one of the most important risk factors for atherosclerosis and its complications. Epidemiological data from the WOBASZ II study indicate that hypercholesterolemia (total cholesterol ≥190 mg/dL or LDL-C ≥115 mg/dL) affects 70.3% of men and 64.3% of women (7). Despite the availability of highly effective lipid-lowering therapies, disease awareness and treatment effectiveness remain alarmingly low. Approximately 60.6% of affected individuals remain undiagnosed, while less than 6% of the population achieve recommended LDL-C targets (6, 7). Examples of digital applications and systems used in dyslipidemia management are summarized in Table 2.

**Table 2:** Examples of digital applications and systems for dyslipidemia management.

Solution	Developer/Country	Functionality	Certification	Clinical Evidence
LDL-C Manager	American College of Cardiology (ACC), USA	Clinical decision support, cardiovascular risk calculator, treatment recommendations	No (clinical support tool)	Implemented in clinical practice; no randomized controlled trials
Corrie Lipids	Corrie Health, USA	Lipid monitoring module, EHR integration	No CE certification in the EU	Pilot studies, feasibility testing
	Academic project, Europe	Patient-oriented application providing reminders, self-monitoring, and follow-up support	None	Pilot studies
My A:Care	Abbott	Lifestyle motivation, adherence support, integration with laboratory testing	None	Pilot studies and survey-based data
CarnaLife System	MedApp, Poland	Integration of lipid profile results with a telemedicine platform	CE-certified medical device	Pilot implementation studies
CardioCube	Poland/USA	Remote patient data collection and monitoring in heart failure	CE-certified system components	Feasibility studies

### Applications supporting physicians in lipid management

One of the most widely recognized tools is **LDL-C Manager**, developed by the American College of Cardiology (ACC). The application enables rapid cardiovascular risk assessment, provides guideline-directed treatment recommendations, and supports clinicians in therapeutic decision-making (24).

Another example is **Corrie Lipids** (26), a module developed within the broader Corrie Health platform. This solution integrates lipid-related data and enables real-time monitoring of treatment outcomes. The underlying study served as a pilot investigation preceding larger clinical trials to evaluate the impact of digital self-management on hard clinical outcomes in secondary prevention following myocardial infarction.

The Corrie Health platform combines a mobile application with wearable technology (Apple Watch) and wireless blood pressure monitoring devices. The application delivers educational content, medication reminders, vital-sign monitoring, and communication tools connecting patients with healthcare providers. The primary objective was to evaluate feasibility, acceptability, adherence, and clinical impact among post-myocardial infarction patients.

More than 90% of participants actively used the application, and medication adherence rates were substantially higher than those typically observed in routine care populations. Users also demonstrated greater engagement in cardiovascular risk-factor management, including physical activity, blood pressure control, and weight management. Importantly, the platform was well tolerated among older adults, while educational support and interactive reminders appeared to reduce medication-related errors.

The authors emphasized that Corrie Health represents an example of a comprehensive digital platform integrating monitoring, education, and communication within a single mHealth ecosystem. Such solutions may improve treatment adherence, enhance clinical outcomes, and reduce healthcare costs through lower rates of rehospitalization following myocardial infarction (25).

### Patient-oriented applications

Among the first digital tools developed specifically for patients is **CoLipid**, a mobile application designed to support self-management by providing reminders for follow-up visits, laboratory testing, and medication dosing schedules (34). Similarly, programs such as **My A:Care** focus on motivating patients to adopt healthier lifestyles, improving treatment adherence, and integrating patient-reported information with laboratory measurements (27).

### Integration with telemonitoring

The previously mentioned MedApp AI CarnaLife System also enables users to enter laboratory results and monitor lipid trends over time (20). Similarly, CardioCube facilitates remote data collection and transmission of patient-generated information to healthcare professionals (28). In the future, laboratory results may become increasingly integrated with wearable devices and mHealth applications, creating more comprehensive digital ecosystems for cardiovascular risk management.

### Gaps in the literature

In contrast to hypertension, the field of dyslipidemia lacks large-scale randomized controlled trials evaluating the effectiveness of digital interventions. Most currently available studies remain pilot projects focused primarily on feasibility, usability, and patient acceptance. Large randomized controlled trials (RCTs) assessing hard clinical endpoints, such as reductions in cardiovascular events, as well as robust cost-effectiveness analyses, are urgently needed.

### Integrated programs for cardiovascular prevention

An important potential application of digital medicine lies in programs that integrate multiple aspects of cardiovascular prevention, including blood pressure control, lipid management, weight reduction, physical activity, and lifestyle modification. The goal of such interventions is to influence the patient's overall cardiovascular risk profile, in line with contemporary concepts of holistic cardiovascular prevention (4). Representative digital programs integrating multiple components of cardiovascular prevention are presented in Table 3.

**Table 3:** Digital programs integrating multiple aspects of cardiovascular prevention.

Program Name	Scope of Intervention	Technology	Clinical Outcomes	Country
PreventiPlaque	Cardiovascular risk assessment and primary prevention based on carotid ultrasound imaging and mHealth data	Mobile application + imaging module (AI-enhanced ultrasound)	Increased risk awareness and lifestyle improvement	Germany
CardioCube	Telemonitoring of post-myocardial infarction and heart failure patients	Mobile application + AI chatbot + EHR integration	Improved adherence and reduced hospitalizations	Poland/USA
Omada Health	Integrated diabetes and cardiovascular prevention program	Online platform + behavioral coaching	Weight reduction, improved glycemic control, and blood pressure management	USA
Comarch e-Care	Remote monitoring of blood pressure, glucose levels, ECG, and physical activity	Telemedicine platform + wearable devices	Improved detection of abnormal values and high patient satisfaction	Poland
MedApp CarnaLife System	Comprehensive telemonitoring (blood pressure, body weight, oxygen saturation, ECG)	Mobile application + cloud platform + data visualization	Earlier detection of disease exacerbations and improved physician–patient collaboration	Poland
HeartHab	Digital cardiac rehabilitation following myocardial infarction	Mobile application + motion sensors	Increased physical activity and improved quality of life	The Netherlands
Multimodal Digital Health Ecosystem (Conceptual Framework)^a^	Integrated monitoring of blood pressure, body weight, body composition, physical activity, sleep quality, nutrition, medication adherence, lifestyle factors, and patient-reported outcomes	Unified digital platform integrating connected devices, patient applications, educational modules, telemonitoring, and clinical decision-support tools	Potential improvement in risk-factor control, patient engagement, adherence, and long-term cardiovascular prevention; currently under clinical evaluation	Poland

a Conceptual framework representing a proposed integrated digital health ecosystem; this model is currently under clinical evaluation and should not be interpreted as an established digital therapeutic.

One relevant example is the previously described PreventiPlaque application, which utilizes visualization of atherosclerotic plaques identified by carotid ultrasound imaging. Patients receive images of their own atherosclerotic lesions, complemented by educational materials and lifestyle-related reminders.

In a randomized clinical trial, this intervention led to greater reductions in SCORE2-estimated cardiovascular risk, LDL-C levels, and systolic blood pressure compared with the control group (29).

Similarly, programs such as Omada Health combine mobile applications, health coaching, and remote monitoring, enabling comprehensive management of lifestyle factors and cardiovascular risk determinants (30).

### Importance of integrated programs

Multicomponent interventions may be particularly useful in cardiovascular prevention because they address several interacting risk factors simultaneously, including blood pressure, lipid management, body weight, physical activity, and lifestyle behaviors. By combining telemonitoring, educational and motivational applications, and active involvement of physicians and nurses, such programs may support sustained behavioral change and long-term treatment adherence (25). However, further randomized and pragmatic real-world studies are needed to determine whether integrated programs provide superior long-term clinical outcomes compared with single-risk-factor interventions and whether they lead to reductions in hard clinical endpoints.

### Telemonitoring in primary care

In both Poland and other European countries, telemonitoring programs are increasingly being implemented within primary healthcare settings. These initiatives typically combine monitoring of blood pressure, blood glucose levels, and body weight. Available evidence indicates that such approaches can significantly improve blood pressure control and glycemic management compared with standard care.

### From single-purpose applications to integrated digital health ecosystems

Historically, most mobile health (mHealth) and digital therapeutics (DTx) solutions have been designed to address a single health problem, such as blood pressure control, physical activity, weight management, medication adherence, or health education. Although these interventions have demonstrated effectiveness in specific domains, a major limitation remains the fragmentation of health data and the lack of integration across different aspects of patient care.

A clear shift is currently occurring from standalone health applications toward multimodal digital platforms capable of integrating data from multiple sources, including connected health devices, electronic health records, patient-reported outcomes, and communication tools connecting patients with healthcare professionals. Such an approach is particularly relevant for individuals with multimorbidity, in whom successful treatment depends on the simultaneous management of multiple interconnected risk factors.

Modern digital health platforms can simultaneously monitor key modifiable determinants of health, including blood pressure, body weight and body composition, physical activity, step count, sleep parameters, dietary habits, medication adherence, substance use, and selected measures of quality of life and self-perceived health status. Integrating these data enables a more comprehensive assessment of health than the evaluation of isolated parameters alone.

In parallel, these systems may provide personalized educational and behavioral support covering healthy nutrition, physical activity, sleep hygiene, smoking cessation, reduction of alcohol consumption, prevention of chronic diseases, and appropriate medication use. The primary objectives of such interventions are to increase patient engagement, improve treatment adherence, and facilitate sustainable behavioral change.

Unlike traditional solutions focused on a single risk factor, more integrated platforms are evolving toward a digital health ecosystem model, in which patients interact within a unified environment integrating health monitoring, education, behavioral support, communication with healthcare professionals, and clinical decision-support functionalities (31). The integration of behavioral, clinical, and environmental data may represent the next stage in the evolution of digital therapeutics, facilitating a transition from reactive healthcare toward more proactive and predictive models of care.

Ongoing interventional studies evaluating multimodal digital platforms may provide valuable evidence regarding the effectiveness of this approach in real-world clinical practice and its potential impact on population health outcomes.

### European and Polish experiences: regulation, implementation, and barriers

**Figure 4:**
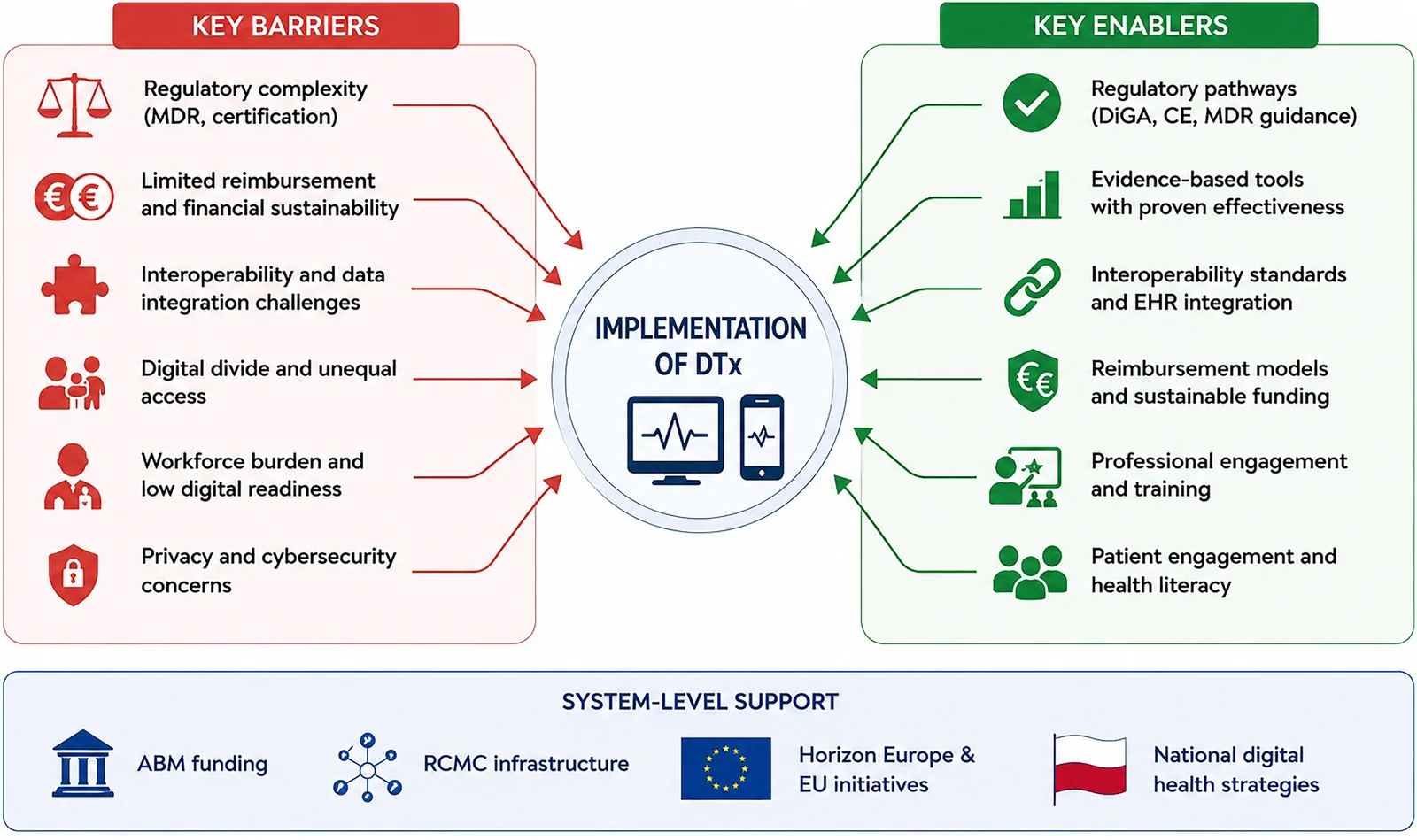
Barriers and enablers of implementing digital therapeutics in cardiovascular care. DTx, digital therapeutics; DiGA, Digital Health Applications (Germany); CE, *Conformité Européenne*; MDR, Medical Device Regulation; EHR, electronic health record; ABM, Agency for Medical Research (Poland); RCMC, Regional Centres for Digital Medicine (Poland).

The regulatory environment and implementation ecosystem for digital health interventions (mHealth and DTx) remain heterogeneous across Europe, influencing the pace of adoption in cardiovascular prevention. Key components include regulatory frameworks (Medical Device Regulation [MDR] and CE marking), reimbursement pathways (e.g., Germany's Digital Health Applications, DiGA), institutions supporting clinical research (e.g., the Polish Medical Research Agency [ABM]), and infrastructures enabling data interoperability and digital transformation, such as the Regional Digital Medicine Centers (RCMC). The principal barriers and enablers of implementing digital therapeutics in cardiovascular care are summarized in Figure 4.

### MDR and CE marking

Regulation (EU) 2017/745 on Medical Devices (MDR) harmonized requirements for medical devices across Europe, including software classified as **Software as a Medical Device (SaMD)**. CE marking confirms compliance with essential safety and performance requirements. For clinical applications, including digital therapeutics for hypertension, MDR requires robust quality management systems, risk assessment procedures, clinical evaluation, and post-market surveillance. In practice, MDR has significantly increased regulatory requirements and extended certification timelines, contributing to the limited number of certified digital health solutions currently available on the European market.

### DiGA: the German reimbursement model for digital therapeutics

Germany was the first country to establish a reimbursement framework for prescription digital health applications, known as **Digital Health Applications (DiGA)**. Applications included in the Federal Institute for Drugs and Medical Devices (BfArM) directory may be prescribed by physicians and reimbursed by statutory health insurers, provided that they demonstrate a positive healthcare effect through clinical evaluation. The DiGA model has accelerated the commercialization of digital therapeutics and facilitated the generation of real-world evidence; however, it requires robust clinical data and strict compliance with data protection standards (32).

### Poland: the medical research agency (ABM), regional digital medicine centers (RCMC), and implementation initiatives

In Poland, the role of the **Medical Research Agency (ABM)** in financing non-commercial clinical studies and digital medicine projects has been steadily increasing. Current funding calls primarily focus on the use of already certified medical devices (CE/MDR-compliant) and the evaluation of their effectiveness under real-world conditions. The emerging network of **Regional Digital Medicine Centers (RCMC)** is intended to provide infrastructure for integrating telemonitoring and mHealth data, standardizing data formats, and supporting multicenter studies in cardiovascular prevention (33).

### Barriers to implementation in Europe and Poland

Several barriers continue to hinder the widespread adoption of digital health interventions:

1. Regulatory and certification challenges. The MDR has substantially increased evidentiary requirements and mandates continuous post-market surveillance. Software developers often face limited availability of notified bodies responsible for certification.
2. Reimbursement barriers—outside Germany, reimbursement pathways remain poorly defined. In Poland, the absence of a DiGA-like framework limits the scalability of DTx solutions within primary care and specialist outpatient settings.
3. Interoperability and Electronic Health Records (EHRs)—standards for integration with electronic health records and healthcare registries are still evolving. Adoption of interoperability frameworks such as IHE (Integrating the Healthcare Enterprise) and HL7 FHIR (Fast Healthcare Interoperability Resources) will be crucial for future implementation.
4. Patient and healthcare professional engagement—maintaining long-term use of digital interventions remains challenging, and additional workload for healthcare professionals may limit adoption. Human-centered design principles and built-in behavioral engagement mechanisms are increasingly recognized as essential.
5. Data security and privacy—compliance with the General Data Protection Regulation (GDPR), data minimization, end-to-end encryption, and consent management are critical, particularly in the context of wearable device data.
6. Workflow integration and clinical responsibility. The implementation of telemonitoring and digital cardiovascular platforms requires clearly defined clinical workflows. Digital tools should not function as isolated patient-facing applications, but should be embedded into routine care pathways with explicit allocation of responsibilities among physicians, nurses, allied healthcare professionals, and technical support teams. In particular, healthcare providers must know who is responsible for reviewing incoming data, responding to abnormal values or alerts, contacting patients, escalating care, modifying treatment, and documenting clinical decisions in the electronic health record. Without such workflow integration, digital systems may increase rather than reduce workload. Excessive or poorly prioritized alerts may contribute to alert fatigue, delayed responses, and uncertainty regarding clinical responsibility. Therefore, successful implementation requires predefined alert thresholds, triage protocols, response times, escalation pathways, and documentation standards. Integration with electronic health records and reimbursement models should also reflect the time and responsibility associated with remote data review and telemonitoring-based care.

### Challenges and future perspectives

Despite the growing evidence supporting digital therapeutics and mobile health applications in cardiovascular medicine, their implementation still faces numerous clinical, technological, organizational, and legal challenges.

### Sustainability of effects and treatment adherence

One of the most significant challenges is maintaining long-term patient engagement. Numerous studies have demonstrated that active use of mHealth applications substantially declines after the first few months of intervention. Addressing this issue requires the development of interventions based on behavioral psychology principles, gamification, content personalization, and behavioral nudging strategies.

### Strategies for enhancing patient engagement in digital therapeutics

The effectiveness of digital health interventions depends not only on clinical content but also on sustained user engagement. Three commonly used approaches are gamification, personalization, and behavioral nudging. Gamification incorporates game-like elements, such as points, levels, rewards, and challenges, to support motivation and short-term adherence. Personalization tailors recommendations, messages, and therapeutic goals to individual characteristics, including age, cardiovascular risk profile, lifestyle, and digital literacy. Behavioral nudging modifies the decision-making environment through reminders, defaults, or context-sensitive prompts that encourage healthier behaviors without restricting patient autonomy.

These strategies may improve engagement and adherence, but their implementation should reinforce intrinsic motivation, avoid excessive profiling, and remain aligned with principles of transparency, privacy protection, and ethical design.

### Data privacy and security

Data privacy and security remain central implementation requirements for digital cardiovascular interventions. Because these tools often process sensitive health information from mobile applications, telemonitoring devices, and wearables, compliance with GDPR, data minimization, encryption, secure consent management, and transparent data governance is essential. These issues are further discussed in the ethical framework section.

### Artificial intelligence and personalized care

Artificial intelligence (AI) has significant potential to personalize digital interventions. Machine learning algorithms may predict cardiovascular events, analyze trends in blood pressure and lipid profiles, and dynamically adapt recommendations based on patient-specific data. Future developments are likely to include AI-powered clinical assistants and decision-support systems designed to assist both healthcare professionals and patients in everyday clinical practice (31).

### Opportunities created by ABM and Horizon Europe

In Poland, the Medical Research Agency (ABM) offers substantial opportunities for funding studies evaluating digital therapeutics under real-world conditions. At the European level, the Horizon Europe framework program provides support for large multinational projects involving telemonitoring, data integration, and innovative AI-based technologies. Such initiatives may accelerate the implementation of digital therapeutics in routine clinical care.

### Economic perspective

From an implementation perspective, economic value remains context-dependent. Although modelling studies suggest that selected digital therapeutics and telemonitoring strategies may be cost-effective, robust evidence of sustained cost savings in routine care remains limited. Future evaluations should distinguish between projected reductions in cardiovascular events and empirically observed changes in healthcare utilization, hospitalization rates, professional workload, and total system costs.

### Ethical considerations and potential risks of digital therapeutics

**Figure 5:**
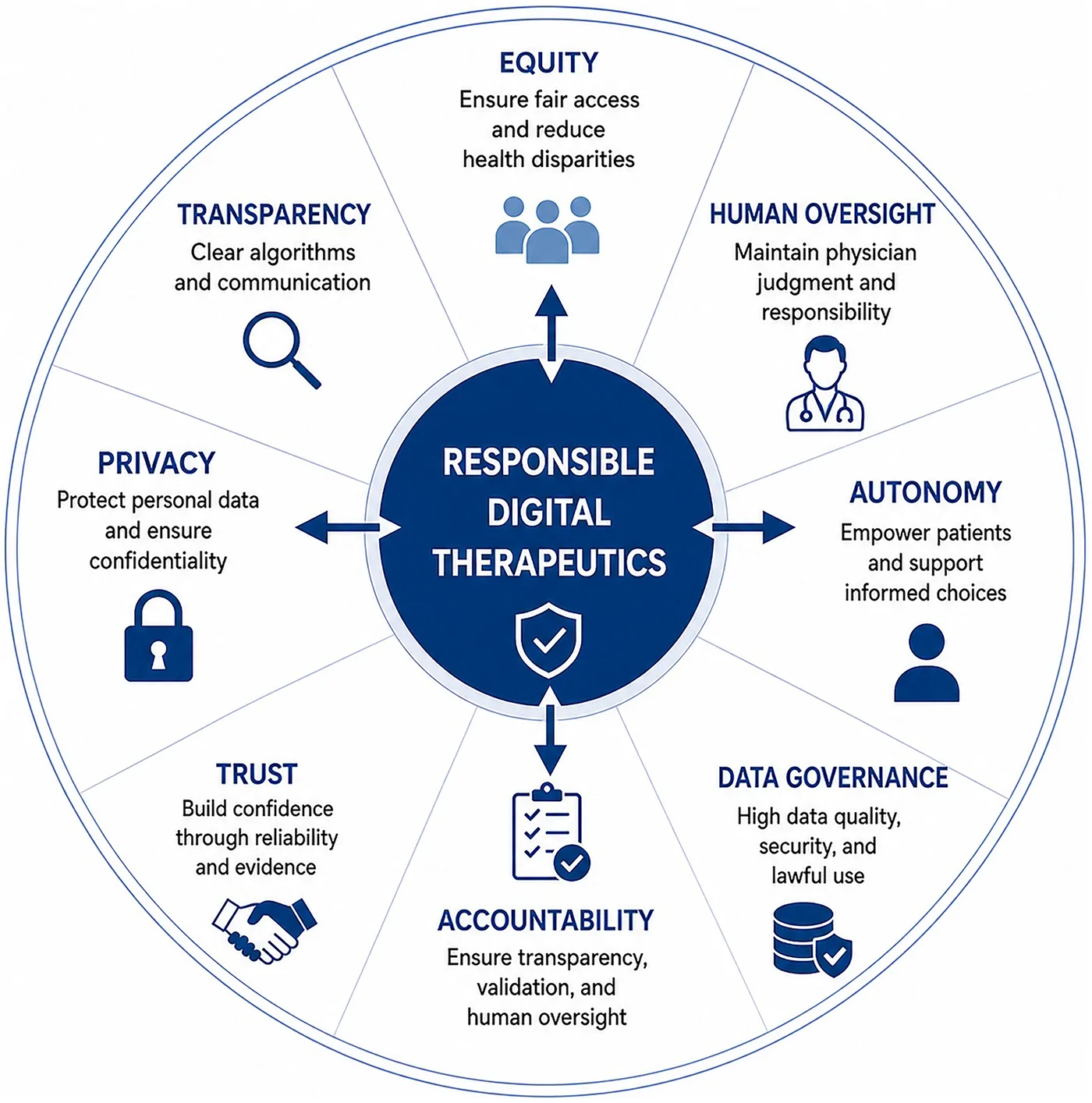
Ethical compass for responsible digital therapeutics. DTx, digital therapeutics.

The key principles for the responsible implementation of digital therapeutics are summarized in Figure 5. The implementation of digital therapeutics in cardiovascular medicine raises several important ethical concerns.

First, digital therapeutics carry a risk of widening healthcare inequalities. Older individuals, patients with limited digital literacy, and populations living in areas with inadequate internet infrastructure may have reduced access to digital interventions, potentially exacerbating existing health disparities.

Second, ensuring data privacy and security is of paramount importance, particularly when information is collected through wearable devices and mobile applications. Such data often include highly sensitive information regarding health status, lifestyle behaviors, and geolocation. Security breaches or unauthorized access may have serious medical, social, and legal consequences.

A further concern involves patient autonomy and clinical responsibility. While digital tools may provide recommendations regarding treatment modifications, final therapeutic decisions should always remain under the supervision of qualified healthcare professionals. Excessive reliance on algorithmic recommendations may result in “overtrust,” while the lack of transparency associated with black-box AI systems complicates validation and accountability.

The potential commercialization of health data also requires careful consideration. The use of patient-generated data for marketing purposes may conflict with the fundamental principle that patient welfare should remain the primary objective of healthcare systems.

From a broader healthcare system perspective, maintaining an appropriate balance between innovation and responsibility is essential. The implementation of digital health solutions should be guided by rigorous clinical evaluation, cost-effectiveness analyses, and assessments of societal impact rather than technological novelty alone. Clear legal and ethical frameworks governing certification, post-market surveillance, and accountability for digital health systems will therefore be necessary.

## Conclusions

Digital interventions are increasingly relevant to cardiovascular prevention, but their evidence base and clinical maturity differ substantially across risk factors and technologies. The most established evidence currently supports digitally enabled blood pressure management, particularly when home blood pressure monitoring, telemonitoring, behavioral support, adherence tools, and clinician-guided treatment adjustment are integrated into routine care.

In contrast, digital tools for dyslipidemia and integrated cardiovascular prevention remain less mature. Their current role is mainly supportive, including patient education, lifestyle modification, medication adherence, shared decision-making, risk communication, and long-term engagement. Although these tools may become important components of preventive cardiology, they should not be presented as established digital therapeutics unless supported by robust clinical evidence, regulatory validation, and clear therapeutic claims.

Future studies should distinguish between digital therapeutics, mHealth applications, telemonitoring systems, digital platforms, and integrated ecosystems. They should evaluate clinically meaningful endpoints, including blood pressure control, LDL-C reduction, treatment persistence, therapeutic intensification, achievement of guideline-recommended targets, cardiovascular outcomes, workflow integration, safety, cost-effectiveness, equity, and long-term sustainability.

### Summary

In summary, digital cardiovascular interventions are most strongly supported by evidence in hypertension, whereas digital tools for dyslipidemia and integrated prevention remain at an earlier stage of clinical validation. Their future implementation should depend on clear terminology, clinically meaningful endpoints, workflow integration, regulatory and reimbursement pathways, data protection, and long-term evidence of effectiveness and sustainability.

During the preparation of this manuscript, the authors used generative AI-assisted tools, including ChatGPT (OpenAI), to support the development of graphical illustrations, figure layouts, visual concepts, language editing, and editorial refinement. All AI-assisted outputs were critically reviewed, verified, revised where necessary, and approved by the authors. The authors retained full control over the scientific content, interpretation of evidence, conclusions, and final editorial decisions, and take complete responsibility for the accuracy, integrity, and originality of the manuscript.

## References

[1] World Health Organization. Cardiovascular diseases (CVDs)—Fact sheet (2025). Available online at: https://www.who.int/news-room/fact-sheets/detail/cardiovascular-diseases-(cvds) (Accessed August 25, 2026).

[2] WHO Regional Office for Europe. Cardiovascular diseases—Fact sheet (Europe) (2024). Available online at: https://www.who.int/europe/news-room/fact-sheets/item/cardiovascular-diseases (Accessed August 25, 2026).

[3] Prejbisz A Dobrowolski P Doroszko A Olszanecka A Tycińska A Tykarski A. Guidelines for the management of hypertension in Poland 2024 — the position of the polish society of hypertension/Polish cardiac society experts. Arterial Hypertension. (2024) 28:91–146. 10.5603/ah.103916 40178258

[4] Visseren FLJ Mach F Smulders YM Carballo D Koskinas KC Bäck M. 2021 ESC guidelines on cardiovascular disease prevention in clinical practice. Eur J Prev Cardiol. (2022) 29(1):5–115. 10.1093/eurjpc/zwab154 34558602

[5] Wierzowiecka M Zieliński M Tykarski A Niklas A. Guidelines are one thing, practice is another. With newer and newer guidelines, why can’t we manage to control modifiable cardiovascular risk factors? Arterial Hypertension. (2024) 28:18–30. 10.5603/ah.99726

[6] Niklas A Marcinkowska J Kozela M Pająk A Zdrojewski T Drygas W. Blood pressure and cholesterol control in patients with hypertension and hypercholesterolemia: results from the polish national health survey WOBASZ II. Pol Arch Intern Med. (2019) 129(10):658–66. 10.20452/pamw.15013 31596271

[7] Pająk A Szafraniec K Polak M Polakowska M Kozela M Piotrowski W. Changes in the prevalence, treatment, and control of hypercholesterolemia and other dyslipidemias over 10 years in Poland: the WOBASZ study. Pol Arch Med Wewn. (2016) 126(9):642–52. 10.20452/pamw.3464 27452484

[8] Stepaniak U Micek A Waśkiewicz A Bielecki W Drygas W Janion M. Prevalence of obesity and overweight among adults in Poland. Pol Arch Intern Med. (2016) 126(9):662–71. 10.20452/pamw.3499 27535012

[9] Polakowska M Kaleta D Piotrowski W Topór-Mądry R Puch-Walczak A Niklas A. Tobacco smoking in Poland in the years from 2003 to 2014. Multicentre national population health examination survey (WOBASZ). Pol Arch Intern Med. (2017) 127(2):91–9. 10.20452/pamw.3896 28224973

[10] Kwaśniewska M Pikala M Bielecki W Dziankowska-Zaborszczyk EŻ Rębowska E Kozakiewicz K. Ten-year changes in physical activity among polish adults: results of the WOBASZ I and II surveys. PLoS One. (2016) 11(6):e0156766. 10.1371/journal.pone.0156766 27272130 PMC4896475

[11] Wang Y Luan Y Cheng S Hao M Tan W Hu R. A multi-layer retrieval-augmented large language model framework for enhancing hypertension education. Hypertens Res. (2026) 49:1428–40. 10.1038/s41440-025-02481-9 41501362

[12] Niklas A Flotyńska A Puch-Walczak A Polakowska M Topór-Madry R Polak M. Prevalence, awareness, treatment and control of hypertension in the adult polish population—multi-center national population health examination surveys—wOBASZ studies. Arch Med Sci. (2018) 14(5):951–61. 10.5114/aoms.2017.72423 30154875 PMC6111367

[13] Kario K Nomura A Harada N Okura A Nakagawa K Tanigawa T. Efficacy of a digital therapeutics system in the management of essential hypertension: the HERB-DH1 pivotal trial. Eur Heart J. (2021) 42(40):4111–22. 10.1093/eurheartj/ehab559 34455443 PMC8530534

[14] Kario K Harada N Okura A. The first software as medical device of evidence-based hypertension digital therapeutics for clinical practice. Hypertens Res. (2022) 45(12):1899–905. 10.1038/s41440-022-01016-w 36207530 PMC9540047

[15] Yoon M Hur T Park SJ Jo SH Kim EJ Kim SJ. Self-monitoring of blood pressure and feedback via mobile app in treatment of uncontrolled hypertension: the SMART-BP randomized clinical trial. Mayo Clin Proc. (2025) 100(5):840–53. 10.1016/j.mayocp.2024.09.018 40047759

[16] Wang Y Guo F Wang J Li Z Tan W Xie M. Efficacy of a WeChat-based multimodal digital transformation management model in new-onset mild to moderate hypertension. Randomized Clinical Trial J Med Internet Res. (2023) 25:e52464. 10.2196/52464 38048156 PMC10728790

[17] Law R. Newel Health obtains EU MDR for blood pressure management platform. Medical Device Network. June 9, 2025. Available online at: https://www.medicaldevice-network.com/news/newel-health-obtains-eu-mdr-for-blood-pressure-management-platform/ (Accessed August 25, 2026).

[18] Newel Health. Amicomed. Available online at: https://amicomed.com/en (Accessed August 25, 2026).

[19] Comarch Healthcare. Comarch e-Care 2.0 telemedicine platform. Available online at: https://www.comarch.pl/healthcare/produkty/zdalna-opieka-medyczna/platforma-comarch-e-care-20/ (Accessed August 25, 2026).

[20] MedApp SA. CarnaLife System and digital medicine solutions. Available online at: https://www.medappai.com/o-nas/ (Accessed August 25, 2026).

[21] Kowalska M Gładyś A Kalańska-Łukasik B Gruz-Kwapisz M Wojakowski W Jadczyk T. Readiness for voice technology in patients with cardiovascular diseases: cross-sectional study. J Med Internet Res. (2020) 22(12):e20456. 10.2196/20456 33331824 PMC7775197

[22] Nomura A Tanigawa T Kario K Igarashi A. Cost-effectiveness of digital therapeutics for essential hypertension. Hypertens Res. (2022) 45(10):1538–48. 10.1038/s41440-022-00952-x 35726085 PMC9474296

[23] Monahan M Jowett S Nickless A. Cost-effectiveness of telemonitoring and self-monitoring of blood pressure for antihypertensive titration in primary care (TASMINH4). Hypertension. (2019) 73(6):1231–9. 10.1161/HYPERTENSIONAHA.118.12415 31067190 PMC6510405

[24] American College of Cardiology. ACC Lipid Manager App. Available online at: https://www.acc.org/Tools-and-Practice-Support/Mobile-Resources/Features/LDL-C-Manager (Accessed August 25, 2026).

[25] Zhang S Zhuang X Lin X Zhong X Zhou H Sun X. Corrie health platform for self-management after AMI. J Am Heart Assoc. (2019) 8(8):e011955. 10.1161/JAHA.119.011955 31020911 PMC6512089

[26] Corrie Health. Corrie Lipids. Available online at: https://www.corriehealth.com/corrielipids (Accessed August 25, 2026).

[27] Abbott. My A:Care. Available online at: https://acarepro.abbott.com/ (Accessed August 25, 2026).

[28] Netizens. CardioCube. Available online at: https://www.netizens.pl/portfolio/cardiocube/ (Accessed August 25, 2026).

[29] Ullrich G Bäuerle A Jahre LM Paldán K Rosemeyer J Kalaitzidis C. Impact of visual presentation of atherosclerotic carotid plaque on cardiovascular risk profile using mHealth technologies. NPJ Digit Med. (2025) 8(1):47. 10.1038/s41746-024-01423-y 39843925 PMC11754887

[30] Omada Health. Available online at: https://www.omadahealth.com (Accessed August 25, 2026).

[31] Zwack CC Haghani M Hollings M Zhang L Gauci S Gallagher R. The evolution of digital health technologies in cardiovascular disease research. NPJ Digit Med. (2023) 6:1. 10.1038/s41746-022-00734-2 36596833 PMC9808768

[32] Bundesinstitut für Arzneimittel und Medizinprodukte (BfArM). DiGA-Leitfaden (Fast-Track)—Version 3.5 (28.12.2023). Available online at: https://www.bfarm.de/SharedDocs/Downloads/DE/Medizinprodukte/diga_leitfaden.html (Accessed August 25, 2026).

[33] Agencja Badań Medycznych (ABM). Regionalne Centra Medycyny Cyfrowej (RCMC). Available online at: https://abm.gov.pl/pl/polska-siec-badan-klinicznych/regionalne-centra-medycyny-cyf/2962,Regionalne-Centra-Medycyny-Cyfrowej-RCMC.html (Accessed August 25, 2026).

[34] Abdullah NAS Mohd Rosli M Abd Aziz ND Mohd Kasim NA. CoLipid: a mobile application for lipid monitoring. Int J Inter Mob Tech. (iJIM). (2024) 18(14):120–9. 10.3991/ijim.v18i14.46827

